# Perceived and Objectively Measured Physical Activity and Sedentary Time among South Asian Women in the UK

**DOI:** 10.3390/ijerph120303152

**Published:** 2015-03-16

**Authors:** Whitney Babakus Curry, Joan L. Duda, Janice L. Thompson

**Affiliations:** 1 School of Health, Sport and Biosciences, University of East London, London E15 4LZ, UK; E-Mail: w.babakus@uel.ac.uk; 2 School of Sport, Exercise & Rehabilitation Sciences, University of Birmingham, Birmingham B15 2TT, UK; E-Mail: j.l.duda@bham.ac.uk

**Keywords:** physical activity, sedentary time, ethnicity, culture

## Abstract

*Introduction*: Limited self-report data suggest that South Asian (SA) women fail to meet physical activity (PA) recommendations. Recent research using objective measures reveals SA women living in the UK have higher PA levels than previously reported, and a pattern of under-reporting PA and sedentary time (ST). There is limited research on SA women’s understanding and experiences of PA/ST, and the cultural contexts and conditions within which they occur. Therefore the aims of this mixed-methods study were to compare perceived PA and ST to objectively measured data and explore PA- and ST-specific contexts, experiences, and sources of PA and ST amongst SA women in the UK. *Methods*: 24 women were purposively sampled to participate in a semi-structured interview from a larger study of 140 women who wore an accelerometer for 7 days. Demographic and anthropometric data were also collected. *Results*: Notable qualitative themes on contextualisation were of adequate PA as “keeping busy” or “being healthy”, and of ST as “lazy” or “resting in old age”. Few participants reported being sedentary, and most believed they were sufficiently physically active. Objectively measured PA/ST indicated that 66% women were less active than perceived (with regard to duration and intensity), with none able to estimate duration of ST. *Discussion*: Findings suggest that overall, SA women have contextualisations of PA/ST that may not coincide with those of researchers, health professionals and policy makers, and lack awareness of the intensity of PA in which they engage and the health risks of high levels of ST. These findings highlight the need for objective measures of PA and ST in this population combined with in-depth qualitative assessments to provide more accurate assessments of these behaviours. This information can subsequently be used to develop health promotion messages and interventions focusing on increasing duration and/or intensity levels of daily activities (e.g., walking, housework) and reducing ST in this population.

## 1. Introduction

Physical activity (PA), defined as bodily movement that expends energy using the skeletal muscles [1], and sedentary time (ST), defined as sitting and laying behaviours requiring ≤1.5 METS [2], are significant independent risk factors for the development of chronic diseases including heart disease, type 2 diabetes and some cancers [1]. The current recommendation for PA accumulation for health benefits is 150 min per week in bouts of 10 or more minutes at a time [1]. Limited research employing self-reported measures of PA (e.g., International Physical Activity Questionnaire, Minnesota Leisure Time Physical Activity Questionnaire) indicates that the majority of South Asian (SA) women in the UK do not engage in the recommended level of PA for health benefits and that they may spend a majority of their day engaged in sedentary activities [3].

A recent systematic review highlighted that 26 quantitative studies (24 using self-report methods, known for inherent recall bias and vulnerable to misinterpretation) and only 12 qualitative studies have been published to date investigating PA among SA women, with none investigating ST [3]. This review broadly indicated that SA women do not engage in the recommended amount of PA, with key themes affecting SA women’s participation in PA including both barriers (e.g., cultural and family responsibilities; caretaking responsibilities) and facilitators (e.g., to improve poor health; Muslim faith). None of these studies focused on SA women’s perceived levels, understanding and experiences of PA and ST, and none compared perceived levels of PA and ST with objective measures. Moreover, qualitative studies have reported seemingly contradictory evidence on SA women’s activity in which some support the notion that SA women do not engage in sufficient PA, with others reporting that they do [4,5,6,7,8]. The limited number of studies and their inherent lack of detail about recruitment, methods, and analyses make it difficult to draw meaningful conclusions about levels of PA and ST in this population.

Due to the limited evidence currently available about the levels, experiences and perceptions of PA and ST among SA women, the aim of this mixed-methods study was to use semi-structured interviews and accelerometry to: (1) compare perceived PA and ST to objectively measured data; and (2) explore PA- and ST-specific contexts, experiences, and preferred sources of PA and ST amongst SA women in the UK.

## 2. Methods

### 2.1. Theoretical Framework

The overarching framework guiding this research was an ecological model of PA and ST that recognises the determinants of PA and ST behaviours being a result of interactions between personal experiences, as well as the social and environmental contexts in which people live [9,10]. This model, in addition to a literature review and aims of the research, helped to inform the development of the semi-structured interview guide. A hermeneutical phenomenology approach was used to guide the qualitative phase of this study [11,12]. The shared experience of being a SA woman in the UK was the phenomenon explored, which involved an examination of participants’ subjective and objective lived experiences related to PA and ST. This approach was used as phenomenological questions ask for meaning and significance and are not designed to “solve” the phenomenon (e.g., change participants’ behaviours); instead, they allow for the examination and interpretation of both commonalities and differences amongst those sharing the experience [12].

### 2.2. Participants and Recruitment

Participants in the current study were recruited from a larger study conducted in Cardiff, Wales, examining PA and ST among 140 SA women (Bangladeshi and Pakistani women) in the UK. from January 2012 to March 2013 [13]. Twenty-four women were purposively sampled based on body mass index (BMI) category (normal, overweight, and obese), self-reported English language ability (fluent/not fluent), and objectively measured PA (low, medium and high) and ST (low and high) levels. BMI and literacy (as an indicator of ability to understand and comprehend interview questions and concepts) were chosen as criteria for sampling based on previous research indicating that these may be important determinants of PA/ST [3,4]. PA and ST criteria were chosen to ensure women from a wide range of activity levels were included in the study. The sample size was guided by Polkinghorne [14], who recommends interviewing five to 25 individuals who have all experienced the defined phenomenon. The sampling strategy was designed to recruit a similar percentage of normal weight (9%), overweight (19%), and obese (81%) women as in the larger study. Demographic and anthropometric data were also collected, including height (to the nearest cm), weight (to the nearest 0.1 kg), waist circumference (to the nearest cm), body fat percentage estimate based on equations validated for SA women (to the nearest 0.1%; BodyStat Quadscan 4000 unit, BodyStat Ltd, Douglas, Isle of Man, British Isles), and postcode to determine Index of Multiple Deprivation (IMD) ranking [15] (the key indicator of socio-economic deprivation in the UK).

### 2.3. Accelerometry

Actigraph GT1M and GT3X accelerometers (Actigraph, LLC, Pensacola, FL, USA) were used to objectively measure PA and ST. Accelerometers are small, non-invasive devices that measure frequency, duration and intensity of bodily movement [16]. A recent validation study reports no significant differences between measurements between these two models of accelerometers, therefore it was deemed unnecessary to conduct further calibration or validation [16]. In order to produce data useful for comparisons with other studies and populations, only uniaxial data were included for analyses [16].

Semi-structured interviews were used to collect data on contextualisation, barriers, facilitators, and experiences of SA women’s PA and ST. The interview guide consisted of 15 questions, with additional probing questions. Questions on the contextualisation of PA and ST included: “What do you think it means to be physically active?” and “What do you think it means to be sedentary?” Women were asked if there were other terms they or others might use instead of “physically active” or “sedentary”. Other questions included those on anything happening at home, in the neighbourhood or community that make it easier or harder to be physically active, what a typical weekday and weekend day is like, and how they and their family/peers view PA/ST. They were asked to recount their daily weekday and weekend schedules. Additionally, questions on PA and ST participation were included, such as: “are you physically active and if so, what activities do you do and how often?” and “are you sedentary and if so, what do you do during those times and for how long?” The interview guide was in English but was orally translated as needed by trained community worker interpreters into Sylheti, Bangla, Urdu, or Punjabi. To verify accuracy of translation, interpreters back translated five interviews from recordings.

Self-reported PA and ST levels were also assessed in the larger study with a sub-sample of 50 women using the short version of the International Physical Activity Questionnaire (IPAQ-SF; [17]) [13]. A total of 6 (25%) of the 24 women interviewed for the present study also completed the IPAQ-SF. The IPAQ-SF is a questionnaire that assesses PA/ST over the previous seven days. Those who reported being fluent in English completed the IPAQ-SF on their own in English. Those with limited or no English fluency completed the questionnaire verbally with the help of a trained translator and in the presence of a researcher.

### 2.4. Interviews

Details on accelerometer data collection and processing from the larger accelerometer study are reported elsewhere [13]. Briefly, women were asked to wear an accelerometer around the waist for 7 consecutive days and to remove it while engaging in water-based activities and sleeping. Due to low levels of swimming among Pakistani and Bangladeshi women in the UK, it is not expected that removal of the device should underestimate this activity [18]. Devices were set at 60-s epochs. Interviews were conducted in participants’ homes or community centres based on their personal preference. Respondents were asked whether they were comfortable participating in the interview in English or another language (e.g., Sylheti, Bangla, Urdu, Punjabi). Upon request, an interpreter was available for translation during the interviews. Some women expressed worry that while they were fluent speaking English, they might not understand all the questions. In these cases, an interpreter was present and assisted when a participant needed clarification at any time. Participants gave verbal and written informed consent. Interviews were conducted by a 30-year-old female doctoral researcher of mixed racial background, and whom has extensive knowledge of Islamic religion and culture (WBC). This researcher has experiences of residing and conducted research in multi-ethnic communities in the USA, UK, and Turkey. All interviews were audio recorded with permission from the participants. Ethical approval for this study was obtained from the University Ethical Review Committee of the University of Birmingham (reference # ERN_12-1316).

### 2.5. Data Analysis

Accelerometer data were downloaded using Actilife 6 software (Actigraph, LLC) and data reduction conducted using Kinesoft software (KineSoft, Loughborough, UK). In order to capture routine activity and variations in activity, three valid days (including at least one weekend day) were included for analysis. A valid day was defined as a minimum of 600 min of registered wear time. Non-wear time was defined as over 60 min of zero counts [13]. Cut points to determine how many minutes were spent at PA intensity levels were: <99 counts/minute (sedentary), 100–1951 counts/minute (light), 1952–5724 counts/minute (moderate), 5725 counts/minute (vigorous), and 9499–∞ (very vigorous) [13]. The IPAQ-SF requires PA to be converted into MET (metabolic equivalent) minutes, therefore for comparison accelerometer data were also converted into MET minutes. One MET is equal to the amount of oxygen consumed at rest, or 3.5 mL of oxygen per kg of body weight per minute [19]. For accelerometer data, moderate intensity PA was derived as minutes of moderate PA × 4, vigorous intensity PA as minutes of vigorous intensity PA × 8, and MVPA as [(minutes of moderate PA × 4) + (minutes of vigorous PA × 8)]. The IPAQ-SF does not estimate light intensity PA. ST for both IPAQ-SF and accelerometer methods are calculated as mean minutes per day [13].

All audio recordings of interviews were transcribed verbatim for analysis by a researcher (WBC) and then checked for accuracy by a research assistant. Deductive content analysis [20] was used to analyse the interviews. Analyses began with repeated readings of the transcripts. Units of analysis were identified as themes that emerged from the literature. During the organisation phase of analysis, these themes were used to develop a categorization matrix into which the data were coded by WBC. Coding was iteratively refined by WBC and JLT using an unconstrained matrix to allow for the creation of additional categories. JLT independently coded 25% (*n* = 6) to check for agreement in coding. A final coding matrix was then developed and all data reviewed again and coded based on the final agreed categories and subcategories. Perceived PA and ST levels were estimated based on frequency, duration, intensity and mode of various activities reported during the semi-structured interviews.

## 3. Results and Discussion

### 3.1. Sample Characteristics

Demographic, anthropometric and objectively measured PA and ST data for each of the 24 interview participants examined in the current study are reported in Table 1. The mean age of this sample was 52.8 ± 10.1 years, mean BMI was 28.4 ± 6.1 kg/m^2^, mean body fat percentage was 49.3 ± 17.7%, and mean waist circumference was 98.8 ± 13.0 cm. This sample engaged in an average of 553.2 ± 59.4 min of ST per day and 34.6 ± 21.5 of MVPA per day. When compared to the larger study sample of 140 women, those participating in interviews were, on average, 6 years older but similar in BMI, body fat percentage, waist circumference, and level of deprivation [13]. 

**Table 1:** Sample characteristics of the 24 South Asian women participating in semi-structured interviews.

^1^ Ethnicity, Age	BMI Category *	Body Fat %	Waist Circumference ^+^	IMD Quintile ^++^	Self-Reported Language Ability	Mean MVPA/Day ^#^	Mean Light Intensity PA/Day ^#^	Mean ST/Day ^#^
(A) Pakistani, 56	Obese	61.06	Unhealthy	1	Fluent	20.07	21.16	567.11
(B) Pakistani, 38	Obese	49.64	Unhealthy	2	Fluent	12.45	24.68	678.9
(C) fiBangladeshi, 52	Obese	53.45	Unhealthy	2	Fluent	64.43	25.84	487.36
(D) Pakistani, 54	Obese	51.31	Unhealthy	2	Fluent	16.86	26.81	603.27
(E) Bangladeshi, 55	Obese	51.63	Unhealthy	2	Fluent	14.32	27.33	605.36
(F) Pakistani, 55	Obese	56.70	Unhealthy	1	Non-fluent in English	11.50	28.82	565.6
(G) Bangladeshi, 63	Overweight	49.48	Unhealthy	2	Fluent	46.42	30.56	437.43
(H) Pakistani, 56	Obese	58.08	Unhealthy	2	Fluent	30.90	33.19	467.39
(I) Pakistani, 66	Obese	53.00	Unhealthy	2	Fluent	32.96	30.24	531.52
(J) Bangladeshi, 67	Obese	61.50	Unhealthy	2	Fluent	7.87	31.06	468.00
(K) Pakistani, 58	Overweight	49.51	Unhealthy	2	Fluent	6.80	58.11	475.21
(L) Pakistani, 60	Obese	56.93	Unhealthy	2	Fluent	37.00	20.12	483.2
(M) Pakistani, 58	Obese	55.76	Unhealthy	1	Fluent	32.54	19.31	499.51
(N) Bangladeshi, 46	Obese	56.66	Unhealthy	1	Fluent	6.32	5.31	643.26
(O) Bangladeshi, 38	Obese	54.54	Unhealthy	2	Fluent	6.50	24.17	616.08
(P) Pakistani, 52	Obese	55.23	Unhealthy	1	Fluent	26.45	22.88	557.34
(Q) Bangladeshi, 62	Obese	57.29	Unhealthy	1	Fluent	17.11	25.17	492.34
(R) Pakistani, 61	Overweight	48.61	Unhealthy	2	Fluent	25.32	12.25	567.83
(S) Bangladeshi, 36	Obese	57.06	Unhealthy	1	Fluent	9.93	9.23	642.27
(T) Bangladeshi, 61	Obese	54.67	Unhealthy	1	Fluent	22.43	21.38	563.12
(U) Pakistani, 60	Obese	53.26	Unhealthy	1	Fluent	19.67	20.11	598.93
(V) Pakistani, 39	Obese	57.10	Unhealthy	2	Non-fluent in English	20.67	17.56	533.8
(W)Bangladeshi, 38	Normal	40.56	Healthy	2	Non-fluent in English	8.33	16.87	622.64
(X) Bangladeshi, 36	Normal	41.23	Healthy	2	Non-fluent in English	10.58	24.29	602.39

**^1^** Participant identification code, * BMI categories as defined by the WHO [18]; **^+^** Healthy waist circumference ≤80 cm, unhealthy ≥81 cm; **^++^** Based on IMD ranks for Wales, 1 = Most deprived, 2 = nearly most deprived [15]; **^#^** reported in minutes.

Five (20.8%) interview participants reported themselves as not fluent in English, and only two (8%) were found to have a BMI value that could be categorised as “normal” based on the World Health Organisation (WHO) cut points for SAs [21]. All women in this study were in the highest two IMD quintiles based on the Welsh IMD rankings, indicating relatively high levels of deprivation [15].

### 3.2. Perceived vs. Objectively Measured PA and ST

#### 3.2.1. Physical Activity

**Figure 1:**
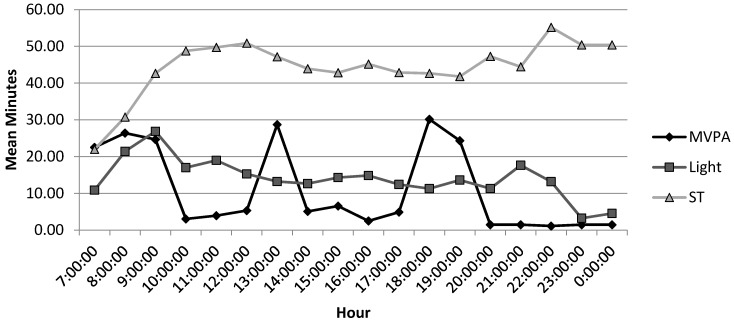
Mean daily ST, light intensity PA and MVPA for 24 South Asian women participating in qualitative interviews.

Since MVPA is a benchmark for activity for health benefits, a comparison was made between reported PA with objectively measured MVPA. Figure 1 shows the mean daily objective MVPA data for the total sample (*n* = 24). The data indicate that during the time periods from 7:00 to 9:00, 13:00 to 14:00, and 18:00 to 19.00, women are the most active. The hour from 18:00 to 19:00 was the most active hour during the day for this sample. As reported in Table 1, on average this sample achieved WHO recommendations of 150 min or more of moderate PA per week (34.6 ± 21.5 min per day). Upon examination, only five (20.8%) participants achieved an average of 30 min or more MVPA per day (range 30.90–64.43 min per day), with 3 achieving 30 min per day in 10-min bouts. Thirteen (54.2%) failed to engage in 20 min or more of MVPA per day. As one of the aims of this study was to compare perceived PA to objectively measured PA across the intensity range, time spent in light PA was also explored. When objectively measured light intensity PA is examined, there is some indication that women are regularly engaging in light intensity PA throughout the day. Light intensity PA was accumulated at an average of nearly 10–15 min of every hour from 9:00 to 21:00 (Figure 1). This light intensity PA may reflect women’s engagement in household activities throughout their day (described in more detail Section 3.5 of the results).

**Figure 2:**
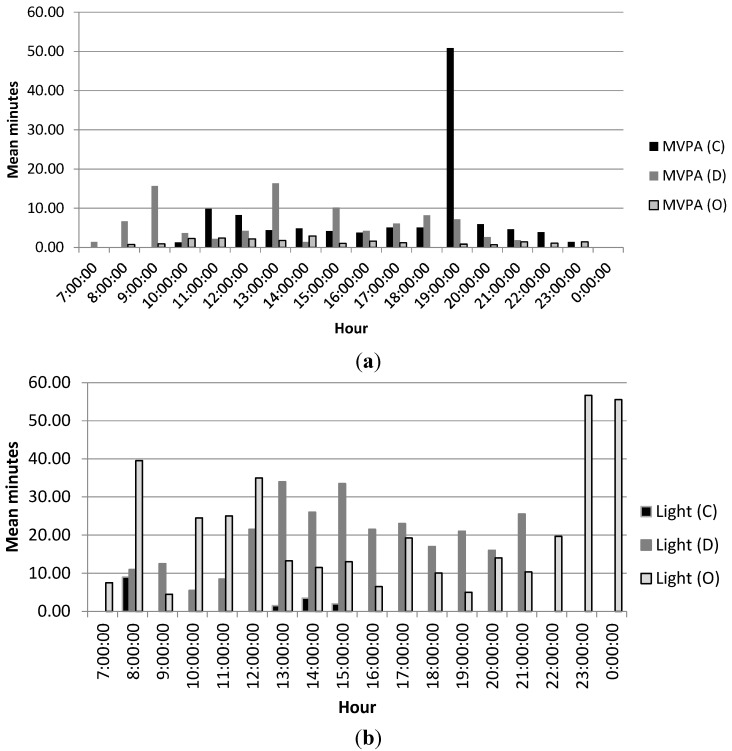
(**a**) Mean daily MVPA for walkers; (**b**) Mean daily light intensity PA for walkers.

Walking was commonly cited as a form of PA in this sample, although only three participants (C, D, and O) reported duration of walking and their results are presented here. Individual results of objectively measured MVPA for interviewees who reported duration of walking can be seen in Figure 2a. Participant C reported walking for an hour per day. As can be seen from her objective data, she achieved 50 min of MVPA in the hour from 19:00 to 20:00. For the rest of the day she accrued minimal MVPA and had only one hour in which she accumulated MVPA in at least 10-min bouts. Participant C’s perceived levels of PA were nearly the same as her objectively measured PA. Participant O reported taking 45-min walks on most days, but her objectively measured MVPA did not accrue above three min at any point in her day. Her perceived levels of PA and her objectively measured levels were drastically different. Participant D reported engaging in 15–20 min of walking in the morning. Objectively measured MVPA for this participant indicates that her perceived level of PA does match with her objectively measured MVPA. She engaged in nearly 16 mean minutes of MVPA in the hour from 9:00 to 10:00. Additionally, she accumulated just over 16 mean minutes of MVPA during the hour from 13:00 to 14:00. She also accumulated a mean 10-min bout of MVPA from 3 to 4 pm.

Figure 2b shows the results of objectively measured light intensity PA for participants C, D and O. Although Participant O did not accumulate 45 min of MVPA per day as she perceived, she did accumulate nearly 60 min of light PA during the hours of 8:00–9:00 and 12:00–13:00, and 50 min of light PA during the hours of 23:00–00:00. While MVPA is the benchmark for health benefits from PA, Participant O’s perceived amount of PA was accurate when taking into account light intensity PA. Participant D engaged in bouts of at least 10 min of light PA throughout her day, indicating her perceived levels of PA does match her objectively measured levels when light intensity PA is taken into account.

**Figure 3:**
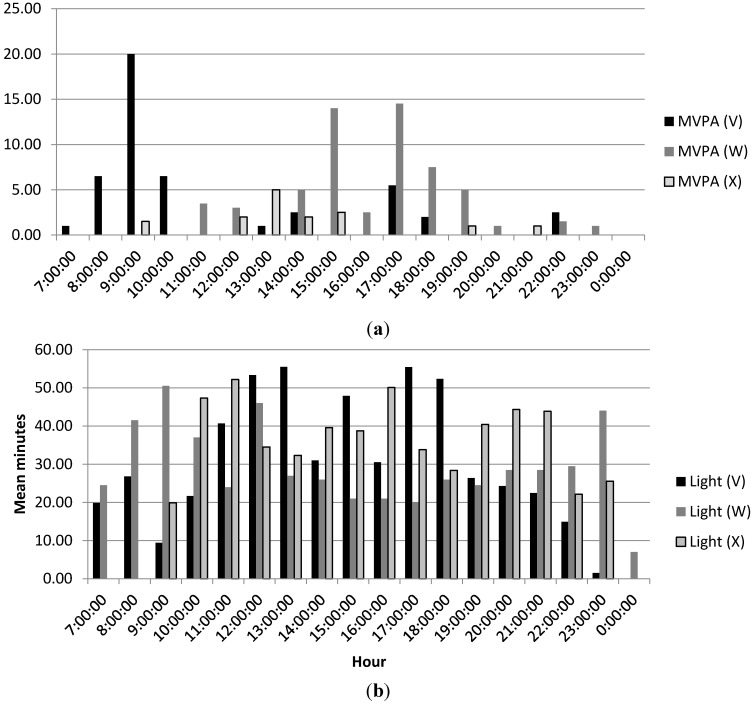
(**a**) Mean daily MVPA for group exercisers; (**b**) Mean daily light intensity PA for group exercisers.

Group exercise class participation was also reported as a mode of PA engagement. Objectively measured MVPA levels for the three women (V, W, and X) who reported that they engaged in group exercise or dance classes during the week are reported in Figure 3a. Participants W and X engaged in some MVPA during the mid-afternoon hours during the week. Since this accumulation does not approach 60 min, it can be inferred that women may not have participated in these classes during the week that activity was objectively measured for this study, that the classes in which they participated did not constitute moderate or vigorous intensity activity levels, or that participants engaged at a lower intensity level during the classes. Participant V engaged in an average of 20 min of MVPA around 9:00, but again this does not seem likely to be the length of a standard group exercise class. Levels of objectively measured light intensity PA for these individuals can be seen in Figure 3b. 

When PA is examined at the light intensity level, participants are much more active at this lower intensity level. In fact, they reach above 50 min of light PA on several occasions throughout the day. A comparison of self-report PA data from the IPAQ-SF with accelerometer data is provided in Table 2. This comparison revealed that three women overestimated the duration of their MVPA, while another three women underestimated the duration of their MVPA when recalling activity over the previous seven days. Women who overestimated MVPA reported they were between 44% and 59% more active than their accelerometer data indicated. Women who underestimated MVPA reported that they were between 32% and 73% less active than their accelerometer data indicated.

**Table 2:** Accelerometer and IPAQ-SF PA and ST comparison for the six participants with both forms of data.

^1^ Ethnicity, Age	Accelerometer MVPA/Day *	IPAQ-SF MVPA/Day *	Accelerometer ST **	IPAQ-SF ST **
(I) Pakistani, 66	694	280	468	480
(M) Pakistani, 58	673	280	492	No recall
(N) Bangladeshi, 46	138	280	605	660
(Q) Bangladeshi, 62	125	520	600	480
(V) Pakistani, 39	161	240	448	660
(W) Bangladeshi, 38	431	240	534	720

**^1^** Participant identification code, * reported in MET minutes, ** reported in minutes.

#### 3.2.2. Sedentary Time 

During interviews participants were unable to quantify their ST during the day. Many responded that they were busy throughout the day with housework, but more sedentary during the evenings. ST appears lower in the earlier hours of the day from 7:00 to 8:00 and then remains constant for the rest of the day, with around 45–55 min of each hour spent being sedentary (see Figure 1). Women’s perceptions that they are not sedentary during the day are not supported by the objectively assessed results. The most sedentary time of day appears to be the evening during 22:00–23:00. This may be a time for reading or television watching, consistent with what respondents reported in interviews. IPAQ-SF data indicate that of the six women who reported ST, two (I, N) were able to recall ST nearly accurately when compared to accelerometer data (Table 2). One woman (M) was unable to recall any ST and two (O, V) overestimated ST compared to accelerometer data. One woman (W) underestimated her ST compared to accelerometer data.

Since few women were able to report their levels of ST during the week, a comparison of their description of activities done during ST was made between women who achieved the highest and lowest levels of objectively measured ST to identify any patterns. When comparing the participant who accumulated the highest ST with the participant who accumulated the least, differences in daily patterns emerged. Participant B had the highest levels of objectively measured ST in the sample. She accumulated less ST around what might be considered traditional times (morning, mid-day and early evening). This could be a time when she was cooking and therefore not sitting down to accumulate as much ST as other times during the day. Participant B says:

“*In the daytime I supposed you know, there [are]…things you can find to do…in the house. There’s always, you know, if it’s not washing, it’s ironing…*”

She also commented on being sedentary in the evenings:

“*…you know, you gotta face the evenings and that’s the worst part of it you know, sitting home and doing nothing.*”

Participant G had the lowest levels of objectively measured ST of the sample. Her levels of ST were lower in the morning from 7:00 to 9:00 and then still relatively low, compared to the total sample, from 12:00 to around 18:00. The interview with this participant revealed that she did housework in the mornings, looked after her 3-month-old granddaughter during the day, and by the evening she was able to sit and relax. After a long day of being busy she notes that:

“*Evening times especially [I am sedentary]. Because I get up early [in] the morning and I’m tired by evening… [I] lie down on the sofa.*”

### 3.3. Conceptualisation and Contextualisation: Physical Activity

Often the terms PA and exercise are used interchangeably in the literature to indicate bodily movement above rest that results in energy expenditure [19]. Therefore the interview began by asking women to explain what each term meant to them before asking how much activity they engage in. Women conceptualised PA as “keeping busy” or “moving around” a lot. This is reflected in the many references to being active through daily housework. As one 36 year-old Bangladeshi woman (S) described:

“*…Physically active to me means doing things. Being busy with life, housework, cooking, cleaning and going out and about.*”

The majority of women (58%) responded that walking was a form of PA and that they did not need to go to the gym to keep active. Some women also responded that being physically active meant to do exercise. Examples of this were going to the gym, doing aerobics, and taking yoga. Women conceptualised the term “exercise” similarly to that of PA, and thus used the terms PA and exercise interchangeably. Many responses included references to walking and housework as PA. Most structured activities such as aerobics, yoga, swimming and going to the gym were repeatedly mentioned as activities that would be considered as exercise. Some women expressed the belief that exercise required “extra effort” and would be “harder” than PA. One Pakistani 52 year-old woman (P) explained:

“*Exercise probably means a bit more in depth, like doing a lot more…Whereas physical activities I just consider like, walking, you know.*”

Another major theme was the idea that PA equates to being healthy. Nearly all women (92%) indicated that being physically active meant being healthy. Particularly, they felt that being active helped keep illnesses away, helped with depression, can lead to weight loss, maintains healthy joints and helps with stress relief. A 39 year-old Pakistani woman (V), when asked what she thought it meant to be physically active, commented that:

“*I think it’s good to be active. It’s good for your health. Um, otherwise you’ve got all these other illnesses that come along.*”

Similarly a 60 year-old Pakistani woman (L) responded that it meant:

“*Good health. Good mind. Help stay away from illnesses.*”

“Good health” was a common response when discussing concepts around PA, and there seemed to be an innate link between PA and health for them. Of particular importance for 38% (*n* = 9) of the sample was the idea that being physically active would help women maintain their independence as they age, and would allow them to be free from relying on others to take care of them by preventing ill health. One 46 year-old Bangladeshi woman (N) stated:

“*…you know, it’s really important to keep yourself healthy because you’ve got to think as well…who’s going to look after you?*”

A 38 year-old Pakistani woman (B) also commented on keeping her independence:

“*Physically active [is] um, that we can do you know, um, all the work* *and can enjoy our life you know, without other people’s help.*”

Remaining free from pain and stiffness was a major concern for the older women who were interviewed. They expressed concern that they would not be able to enjoy doing the things they wanted to without staying active. For example, a 58 year-old Pakistani woman (K) said:

“*Physically active means whatever you want to do, you are able to do it without ah, feeling you’re ah, you know, you’re in pain or anything like that.*”

### 3.4. Conceptualisation and Contextualisation: Sedentary Time

The concept of “being sedentary” was explored before asking participants how much ST they engaged in. Twenty-one women (87.5%) did not know what the word sedentary meant and required an explanation by the interviewer. Once defined (as spending free time sitting or laying down), participants expressed the concept of being sedentary as falling into two main categories. Firstly, it was seen as resting or “taking it easy.” Nearly all women who referred to being sedentary as resting commented on a sense of deserving a rest after a long and busy day. Many women wanted to relax after doing their housework. One 61 year-old Pakistani woman (R) said:

“*I have um, something…to do all the time. Either cleaning the house or like you know…Well in the evening I do take rest. I just um, I lay down and then I do nothing. But um, I think that I deserve [to rest] after the whole day.*”

Other women referred to being sedentary as being lazy. These women conceptualised being sedentary as not keeping busy, not keeping up with their housework and being a “lady of leisure.” Importantly, both younger and older women remarked that after a woman becomes a grandmother (around the age of 40 years), she becomes very sedentary and stop doing things for herself. This was reported as being a widespread phenomenon in the community. The daughter-in-law was identified as being primarily responsible for taking care of the older woman and what used to be her household duties. One older (60 year-old) Pakistani woman (L) commented:

“*…problem becomes when woman becomes mother-in-law and comes home and that’s it. It’s my time to sit now. She [daughter-in-law] will do everything. It’s her responsibility. I done my job. Even mother-in-law may still be in early forties. So that’s where the thing goes wrong. Personally that’s my view.*”

One younger (36 year old) Bangladeshi woman (X) agreed, and expressed the difficulty in changing this cultural norm:

“*They [daughters-in-law] have to encourage their mother-in-law to do a little bit of housework. Um, I know like within Asian culture it’s rude to ask somebody to do that.*”

Additionally, 75% of women (*n* = 18) stated that being sedentary either exacerbated their current illnesses and injuries or could bring about new ones. Obesity, diabetes, depression, and limb pain were the most common conditions mentioned in interviews.

### 3.5. Sources of PA

Twenty-one (87.5%) women reported being physically active. Of these women, 85.7% (*n* = 18) said that they did so by doing housework throughout the day. This included tasks such as cooking, cleaning, vacuuming, and ironing. None reported a specific amount of time spent on housework, but broadly indicated that it was done throughout the day and in between prayer and meal times. Younger women who were not yet grandmothers reported that they were able to stay active because they were busy taking care of their children in addition to housework. One 38 year-old Bangladeshi woman (O) recounted her busy day:

“*Ok. I wake up in the morning and get the kids ready, make their breakfasts. Um, then when I take them to school, come back home, clean the house, do the cooking. Um, after that you know, when the cooking is done, by that time the kids are home. I pick ‘em up. I drop them off. I pick them up. Getting them ready again to go to the mosque. Feeding them. And then when it’s evening time um, when they’re in the mosque, the little ones are there so I’m either doing ironing, or something, I’ll be doing. Just to get all the housework out of the way. And um, and when the kids come home, bedtime, getting them ready, bathing them and bedtime. And um, in the mornings, sorry sometimes when I don’t have housework and um, I don’t have um, what’s it called, um, cooking to do then I’ll do my business. Sit down and do some calls. So I am active every day. And when they go to bed I’m like too tired now and I want to go to bed (laughs).*”

Many women reported similar daily schedules and reported this pattern of being busy as being physically active. Even those who did not report currently being active stated that doing more housework would keep them active, but they did not make a connection between the types of activity they engage in and the intensity at which they perform them.

Taking care of children was not the only source of activity for women; 29.2% (*n* = 7) of women reported being active through caring for another family member. This often included a mother-in-law, father-in-law, and/or grandchildren. One 52 year-old Pakistani woman (P) spoke about her many responsibilities:

“*I’m a housewife. So [I’m] busy in the home. Because we are Asian, Pakistani. I have three sons grown up. And um, husband has not been very well. He was engineer but he can’t work because he’s so ill. Um, and I have a mother-in-law. Ya, she’s old. She’s over 70. My father-in-law passed away about seven years ago so then I look after my mother-in-law. And my children, my husband.*”

Eight (33%) women reported that they currently walk for PA. As previously described, only three women were able to recall the length of time they walked. One woman reported walking for 15–20 min per day, one stated she walked for at least 45 min per day, and another stated she walked for 1 h per day. Four women reported that they were active when they went shopping during the day, but did not report the length of time or intensity for this activity or how many days per week they engaged in shopping. Three women cited group exercise classes as another way of staying active. These classes included Extend (seated or standing light exercise class), Zumba (or other dance class), and yoga. One 36 year-old Bangladeshi woman (S) recalled:

“*…with my friends…every week we get together. So after our chit chat, food and everything we do …at least we do Zumba. That could be sometimes two, three times a week*.”

None reported the duration of the classes, although based on observations of classes held in community centres in the area, these classes typically last for approximately 1 h. One woman stated that she took a class two days per week, while the others did not specify frequency. Three women recalled that they went to the gym to stay active, although none specified what types of activities they engaged in at the gym. One woman, the same woman who recalled two days of group classes, reported 1 day per week of going to the gym.

### 3.6. Sources of ST

Two women (8.3%) in the sample reported being sedentary. The favoured activity of women who reported being sedentary was watching television. This most commonly took place in the evenings, after dinnertime. Few participants (17%) reported how long they watched television per day. One 38 year-old Bangladeshi woman (W) describes why television may be so attractive to SA women:

“*…these days um, everybody, every community has their own channel [referring to South Asian TV channels in native languages]. Which means they are being lazy. Because um, there’s no um, language barrier and they just watch their language channels. They understand them. They enjoy it. So that means um, after lunch or after… they just sit there and…as a family and watch TV. Which is again, not good.*”

Other popular activities done while sedentary were reading and attending prayer groups with friends. During these times women were engaging in religious readings or prayer by themselves or with friends. Women did not report the duration of these activities but broadly indicated that they were afternoons away from the home or during quiet times in the evenings. Results from objective measures conducted with this sample of 24 women indicate that on average this sample of women engaged in 553.2 ± 59.4 min of ST per day (range = 437.4–678.9 min per day). Individual results for the women who participated in the interviews are reported in Table 1. 

### 3.7. Discussion

This mixed-methods study offers useful insights into the perceptions, contextualisations, and sources of PA and ST amongst a small sample of SA women in the UK. It is important to emphasise that because the sampling strategy was designed to recruit a similar percentage of women who were normal weight, overweight and obese as found in the larger study (and as defined by the WHO for SA adults [21]), this resulted in only two women of normal weight participating in interviews. Thus, these results are predominantly reflective of the views of the obese women who participated in the study. Overall, findings suggest that SA women have conceptual understandings and contextualisations of PA and ST that may not coincide with those of researchers, health professionals and policy makers. Broadly, perceived intensity levels of PA and ST, and conceptualisations of what comprises “adequate PA” differ from levels of objectively measured PA and ST in this group. However there was some congruence amongst a small number of participants. Additionally, these results provide unique insights into common sources of PA and ST amongst this under-studied population, along with confirming previously reported barriers and enablers to PA and ST amongst ethnically diverse women. These findings not only add to our knowledge about factors that may be influencing levels of PA and ST in SA women (and in particular, obese SA women), but may also be of use when planning and implementing interventions to increase PA and decrease ST.

#### 3.7.1. Comparison of Self-reported Perceived *versus* Objectively Measured PA and ST

Although the interview data indicated that participants assumed they were sufficiently active (via regular housework and domestic responsibilities), only 20.8% (*n* = 5) of women actually met recommended guidelines for PA adequate to achieve health benefits. Of the six women who were able to quantify their daily PA, two women’s perceived PA matched the MPVA data obtained from objective measures. However, when examining levels of light intensity PA, all six women were as active as they perceived. This finding highlights the importance of clearly defining and emphasising the intensity level of PA needed to achieve health benefits. SA women may perceive themselves as adequately physically active, but may not be meeting recommendations due to their engaging in a lower than adequate level of activity intensity. As most of these women lead busy lives, and they equate this “busy-ness” to being sufficiently physically active. This finding, in addition to perceiving that engaging in housework is sufficient to meet PA guidelines, identifying lack of time for PA due to prioritisation of household and family obligations, and concerns about personal safety are consistent with studies on SA women and research with other ethnic minority women [8,22,23]. New insights gained from our study highlight the unique details of SA women’s lives that are embedded within these broader themes, including:

(1) prioritising traditionally cooked meals from scratch each day, which takes 2 to 3 h on average;
(2) perceiving prayer time (typically three to five times per day) as adequate duration and intensity of PA;
(3) abrupt shift in how time is spent once a woman becomes a mother-in-law; and
(4) engaging in sufficient duration of PA with little experience or insight into how to exercise at an intensity that is moderate or vigorous.

When developing future health promotion and interventions aimed at this population, it is critical to emphasise the intensity of activity that is likely to result in health benefits, and to provide real-life examples of moderate and vigorous intensity activities in which women can engage. As such, health promotion messages and interventions should not assume that this population is by default inactive, and in response simply promote “doing more”. More consideration should be given to working with this population to explore both the amount and intensity of current PA, and tailor efforts toward optimizing both of these components. As researchers and health professionals, we assume that our public health messages and clinical advice is clear and adequate, but these findings suggest this may not be the case.

The majority of women in the targeted sample did not recall specifically when they participated in, or the duration of, their ST. However, they could easily recall what activities they engaged in while being sedentary. Television viewing and reading were the most common forms of sedentary behaviour. While activities such as television viewing have been found to be correlated with low PA and high ST in SAs in the UK [24,25], this is the first study to date to report this as a preferred sedentary activity of SA women. The total sample accumulated an average of nearly six h per day of ST, which is similar to the findings of the Health Survey for England 2012 for the general population [26]. When examining the sample as a whole, women appear to be sedentary for the majority of every hour during the day from 7:00 to 00:00. But when individual activity patterns are investigated, some variation is observed. The times around meals are less sedentary for some women. This is most likely due to women getting up and moving about to prepare and serve meals. Other women were slightly less sedentary during each hour throughout the day, reportedly due to the constant housework that many women cited as a major part of their daily routines. Many women perceived that they were not sedentary due to their “busy” schedules, but objective data revealed that 20 (83.3%) of the women in this sample accrued at least eight h per day of sedentary time. These findings are comparable to those from the few published studies on SA women in which self-reported PA and ST were assessed [8,23]. Future health promotion interventions should aim to encourage women to engage in less sitting time, and break up their bouts of sitting time, throughout their day, even if they are unable to participate in more PA. Findings indicate that tasks such as housework or preparing meals may reduce time spent being sedentary for the SA women in this study. Such activities could convey health benefits independent of MVPA [27].

Additionally, IPAQ-SF and accelerometer data comparisons indicate that women had difficulty recalling PA and ST. While this is a small sample and the results cannot be generalised to all SA women in the UK, this finding demonstrates the difficulties faced when using self-report tools to quantify PA and ST. Such findings are consistent with other studies on ethnic minority groups and confirm the need for an updated culturally tailored self-report assessment tool with strong validity and reliability that can be used if objective measures are not feasible [28,29].

#### 3.7.2. Contextualisation of Physical Activity and Sedentary Time

SA women in this study conceptualised PA first and foremost as being good for their health. This was the most salient concept for them, perhaps because of the very high prevalence of chronic disease, disability or injuries among this group. Nearly all women reported having at least one of these conditions, and nearly all women responded with “good for health” when asked what PA meant to them. When probed for what types of activities might constitute PA, most identified housework and “keeping busy” as their main modes of PA. Although previously identified as one of the means by which people can accrue moderate levels of PA, more recent research indicates that housework may not contribute to reducing conditions such as CVD and obesity, although it has been found to be associated with lower all-cause mortality [27]. This has important implications if SA women are assuming that their housework will give them health benefits such as managing diabetes or reducing CVD and obesity.

The majority of women reported not knowing what the word “sedentary” meant. After the researcher defined the term as “spending free time sitting or laying down”, women provided one of two conceptualisations of this term: Resting or being lazy. Since most women perceived their days to be filled with housework and family obligations that kept them busy, many believed that they deserved to rest in the evenings. This was most often done while sitting and watching television. In contrast, women saw it as being lazy if a woman was known to be “sitting around”. If this was the case, she was not completing her household tasks and was therefore viewed negatively by others in the community. The only exception to this negative opinion was in reference to mothers-in-law. It was an accepted fact that once a woman is a mother-in-law, she was entitled to do a great deal of sitting after having raised a family and done the housework for many years. At this point in the life course, the daughters-in-law take over these responsibilities. While everyone acknowledged this belief, both the younger (not yet mothers-in-law) and older (mothers-in-law) women conceded that this habit was not healthy to engage in. All women recognised that there are health risks that result from leading a sedentary lifestyle. The important influence of family traditions has been found in other studies on SA women [8,23,30]. These studies also suggest that women understand the importance of being active, but that family responsibilities and traditions often prevent them from engaging in PA.

These concepts and contextualisations have important implications for the use of self-report methods in assessing PA and ST among SA women. Previous research indicates there may be issues with SA women’s interpretation of certain terms and concepts from self-report questionnaires, such as what is actually being captured within the item content of the International Physical Activity Questionnaire (IPAQ) [13]. The findings from this mixed-methods study illustrate that while women recognise the general benefits of PA and risks of ST, they may have a very narrow understanding of the meaning of each term and a limited knowledge of what activities constitute adequate PA or inappropriate amounts of ST. It is even more challenging for women who have limited or no English language skills to understand and apply these definitions and concepts to their daily lives.

One major strength of this study is the measurement of both objective measures of PA and ST and participants’ perceived levels from interviews. This approach allows for comparisons and convergence of the two methods. It provides detailed insights into cultural contextualisations of behavioural patterns of PA/ST throughout the day that objective measurement neglects. This includes insights into SA women’s understanding and description of PA/ST in their lives, of which objective measures cannot provide. The use of interviews rather than highly structured questionnaires as a method of collecting PA/ST data allowed for an in-depth exploration of SA women’s own understanding of their activity levels. This study demonstrates not only that SA women may not accurately recall their levels of PA or ST, but provides insights into why this might be the case. Inaccurate recall of PA and ST levels has also been reported in other studies with both ethnic minority groups and the general population [31,32]. While self-report methods of assessing PA and ST are popular due to their ease of use and low cost, they may not accurately reflect actual PA and ST among SA women. It is recommended that not only should objective measures be more widely used in this and other comparable populations, but that they should be used in conjunction with self-report methods to more comprehensively measure PA and ST and gain further insight into how culturally diverse groups understand and view when and where they engage in these behaviours.

Another advantage of the comparison of perceived and objectively measured PA/ST performed in this study is that it results in a more in-depth understanding of the types and levels of intensity of activities that SA women currently engage in. The commonly held belief that housework is an activity that helps a person to achieve an appropriate level of PA for health benefits may not be the case if it is not performed at a moderate-to-vigorous intensity level for sufficient periods of time. SA women may believe that they are sufficiently physically active due to their busy schedules and their engagement in light intensity PA, and hence may not recognise the importance of doing additional or higher intensity activity. Health promotion intervention developers can benefit from this insight and build upon the light intensity activities that women currently engage in to promote increasing the intensity of activities performed to achieve the recommended levels of MVPA. A further strength of this study was the inclusion of non-English speaking participants, as these individuals are often identified as “hard to reach”, can be viewed as difficult to recruit, and as a result are excluded from research.

This study is not without limitations. By design, the sample is small and is not representative; therefore generalisations about these findings must be made with caution. Purposive sampling was used in order to gain a range of perspectives among SA women since it was not possible to collect and analyse data from a much larger sample within the scope and timeframe of this study. The majority of the women who participated in this study were obese, and were recruited from community centres where many activities are offered for SA women. Consequently, the views of women of normal weight were not fully represented. Additionally, participants were more likely to know what is available in their local area, as well as potentially more likely to know about and participate in PA, than SA women in the general community. Although the high number of obese women interviewed can be viewed as a limitation, we feel this might also be considered a strength, as this is the group that is the highest priority to target for intervention and are considered “hard to reach”. Interview questions about PA and ST patterns did not probe specifically into intensity and duration; however, a comparison of objective PA and ST data with self-reported data using the IPAQ-SF from the larger study in this population indicated that participants significantly under-report both their PA and ST [13]. The results from the present study employing in-depth interviews enhance our understanding of SA women’s perceived levels of PA and ST, which is not possible using accelerometry or quantitative self-report tools.

It is also important to recognise the role of the researchers in interpreting the accounts of the SA women in this study. Researchers bring their own presuppositions and distinctive perspectives into their research, and it is necessary to acknowledge that these can have an influence on the interpretation and understanding of data, results and conclusion [33]. The researcher (WBC) who collected, analysed and interpreted the data for this study was a 30-year-old female with a mixed racial background. Another researcher (JLT) who also analysed and interpreted the data was a white female researcher in her 50’s. Both researchers have lived and conducted research in ethnically diverse communities in the U.S. and UK, and they also have experience conducting research with Muslim women in the UK, Turkey (WSB), and Bangladesh (JLT). Despite their experience and knowledge, the demographic and cultural backgrounds of the researchers may have influenced the way in which the data were interpreted and indeed even the research design and methods of this study [34,35]. Therefore the findings presented in this study should be considered with caution due to inherent researcher bias.

## 4. Conclusions

Findings from this study suggest that self-report methods of measuring PA and ST among SA women may not produce accurate data due to participants’ misunderstanding of those terms. Therefore it is recommended that objective measurement of PA and ST be used more widely in this group. Since this is not always possible due to cost and time constraints, it is suggested that a more culturally appropriate self-report questionnaire be developed to improve the accuracy of PA and ST data [36,37]. While interpretation of the current results must be cautious due to the small sample size, our findings suggest that some SA women may only need to increase their activity intensity in order to reach MVPA guidelines. Future interventions and health promotion programmes should focus on helping SA women to understand what engaging in moderate and vigorous intensity activity feels like, and to encourage them to engage at that intensity as much as possible in their daily activities. It is also important that SA women are informed about the health risks associated with high levels of ST, and are provided with realistic strategies to increase PA intensity and break up ST. Increasing PA and decreasing ST in this population will require culturally competent health promotion, interventions and health practitioners that are aligned with participants’ perspectives and cultural understanding. Moreover, practitioners and policy-makers will need to work together with community groups and lay leaders to encourage engagement in health-promoting active lifestyles in SA communities. 
